# Does preoperative mechanical prophylaxis have additional effectiveness in preventing postoperative venous thromboembolism in elderly patients with hip fracture?—Retrospective case-control study

**DOI:** 10.1371/journal.pone.0187337

**Published:** 2017-11-09

**Authors:** Ji-Hoon Nam, Dae-Hwan Kim, Je-Hyun Yoo, Ji-Hyo Hwang, Jun-Dong Chang

**Affiliations:** 1 Department of Orthopaedic Surgery, Hallym University Sacred Heart Hospital, Anyang, Republic of Korea; 2 Department of Orthopaedic Surgery, Kangnam Sacred Heart Hospital, Seoul, Republic of Korea; 3 Department of Orthopaedic Surgery, Dongtan Sacred Heart Hospital, Hwasung, Republic of Korea; Holbæk Hospital, DENMARK

## Abstract

**Background:**

Elderly patients undergoing hip fracture surgery (HFS) are at increased risk of postoperative venous thromboembolism (VTE). To reduce this risk, combined postoperative mechanical and chemical thromboprophylaxis has been routinely performed after HFS in these patients. This retrospective case-control study was conducted to evaluate the additional effectiveness of preoperative mechanical thromboprophylaxis for the prevention of VTE following HFS in elderly patients.

**Methods:**

Of 539 consecutive patients aged 70 years or older undergoing HFS, 404 (control group) did not receive preoperative mechanical thromboprophylaxis, while 135 (study group) received mechanical thromboprophylaxis using an intermittent pneumatic compression device and graduated compression stockings from the time of admission until surgery. All patients received combined postoperative mechanical and chemical thromboprophylaxis following HFS in accordance with the same protocol. The incidence of symptomatic VTE confirmed based on clinical symptoms and 3-dimensional CT angiography within one month of surgery was investigated in both groups.

**Results:**

The American Society of Anesthesiologists grade was higher (p = 0.016) in the study group and more patients in this group had concomitant cardiovascular and neurologic diseases (p = 0.005 and p = 0.009, respectively). In addition, more patients in the study group had received anticoagulant medication preinjury owing to comorbidities (39% vs 28%, p = 0.025). The overall incidences of symptomatic deep vein thrombosis (DVT) and pulmonary embolism (PE) were 7.4% and 3.7%, and 2.2% and 1.5% in the control and study groups, respectively. According to multiple logistic regression, symptomatic DVT significantly reduced in the study group (OR 0.28, p = 0.042), while there was no significant difference in the incidence of symptomatic PE between the two groups (p = 0.223).

**Conclusions:**

Preoperative mechanical thromboprophylaxis may confer an additional benefit by preventing postoperative VTE without adding more risk of perioperative bleeding in elderly patients with hip fracture.

## Introduction

Elderly patients undergoing major orthopaedic surgery including hip fracture surgery (HFS) are at increased risk of postoperative venous thromboembolism (VTE) because of reduced mobility, multiple medical comorbidities, and recent trauma and/or surgery [1,2]. Moreover, elderly patients, aged over 70 years, are at higher risk of VTE because of their advanced age in addition to risk factors listed previously [2–4]. VTE following HFS in these patients is likely to lead to serious morbidity and mortality [5,6]. Therefore, greater attention should be paid for preventing VTE in elderly patients undergoing HFS.

For patients undergoing HFS, the 9^th^ edition of the American College of Chest Physicians (ACCP) guidelines recommends low-molecular-weight heparin (LMWH) therapy as the first drug for preventing postoperative VTE [2]. Mechanical prophylaxis is recommended in cases in which pharmacological therapy is contraindicated or patients who are at high risk of bleeding, or as an adjuvant of anticoagulant therapy. Intermittent pneumatic compression device (IPCD) and graduated compression stockings (GCSs), used in mechanical prophylaxis, enhance venous drainage and prevent venous stasis in the lower extremities, enhance fibrinolytic activities, and reduce the risk of deep vein thrombosis (DVT) [7,8]. Mechanical thromboprophylaxis is effective and safe in most patients because it carries little risk of bleeding or hematoma formation in postoperative patients. However, there are a few contraindications, including dermatologic diseases and severe peripheral arteriopathy [9,10]. Recently, several authors have recommended combined chemical and mechanical thromboprophylaxis to both maximize the efficacy of therapy and reduce the risk of bleeding [2,11].

Fragile, elderly patients with hip fracture are at high risk of VTE. HFS in these patients is often delayed because of comorbidities and their associated routine evaluations [12,13]. The risk of VTE in these immobile, elderly patients may increase during the preoperative period. Although ACCP guidelines recommend initiating LMWH therapy preoperatively in case of delayed surgery, there is little information in the current literature about routine preoperative thromboprophylaxis and its effectiveness in elderly patients with hip fracture. Preoperative initiation of anticoagulation therapy can reduce the risk of VTE; however, it may increase the bleeding risk intraoperatively and postoperatively [14,15]. Several authors reported no difference in the effectiveness of preoperative versus postoperative initiation of anticoagulant therapy for VTE prophylaxis following major orthopaedic surgery [5,16].

Although preoperative mechanical prophylaxis in addition to routine postoperative thromboprophylaxis, could be more beneficial without additional bleeding risk in terms of preventing VTE, the additional effectiveness of mechanical prophylaxis during the preoperative period in elderly patients undergoing HFS is still unclear.

The aim of this retrospective case-control study was to evaluate the additional effectiveness of preoperative mechanical thromboprophylaxis on the prevention of postoperative VTE in elderly patients, aged ≥70 years, with hip fracture.

## Materials and methods

### Study subjects

**Fig 1:**
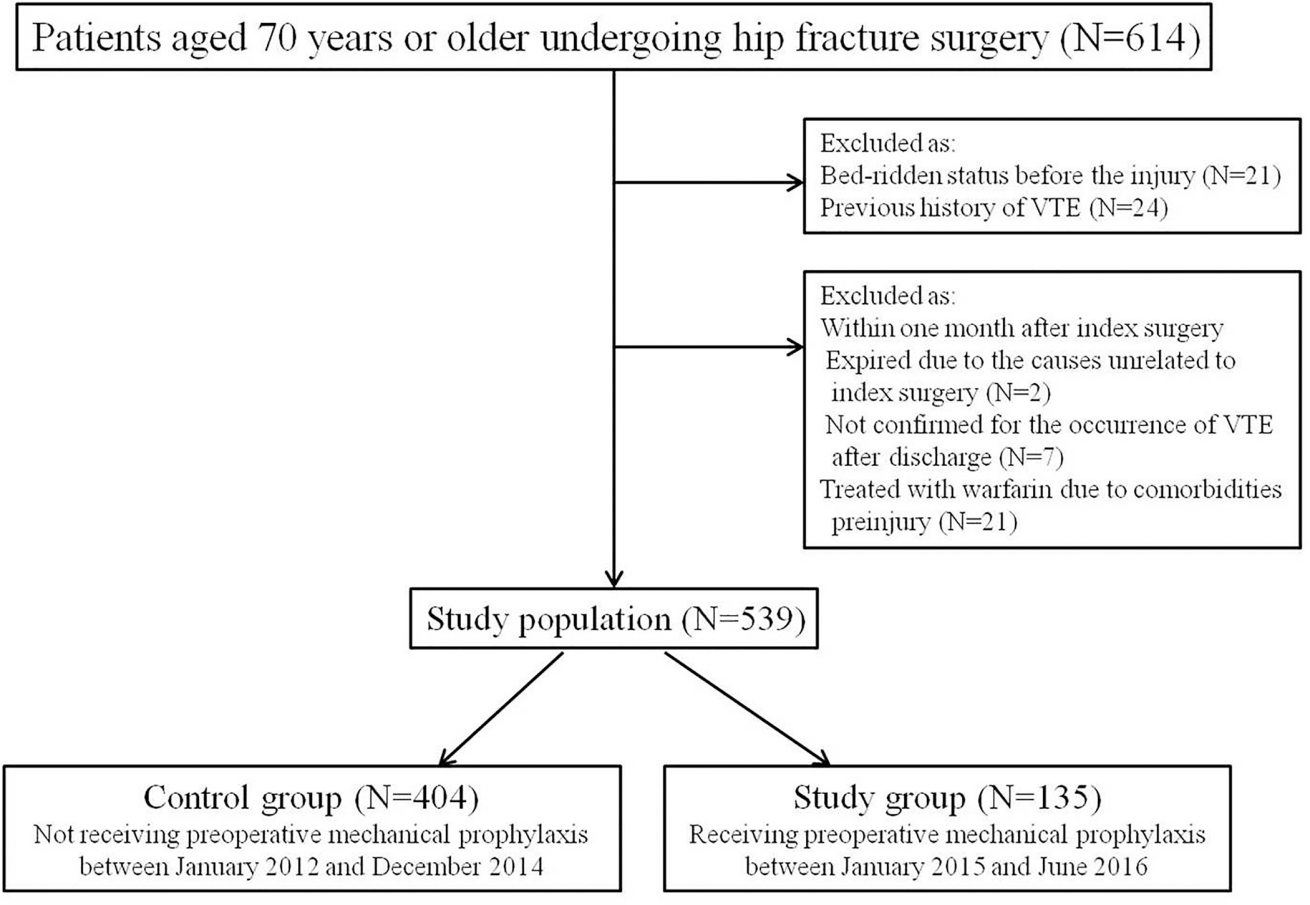
Flowchart demonstrating patient selection and exclusions.

We retrospectively reviewed 614 consecutive patients over 70 years of age, who underwent surgery for a femoral neck or intertrochanteric fracture, between January 2012 and June 2016, in a single university hospital. These patients underwent intramedullary nailing for an intertrochanteric fracture and bipolar cementless hemiarthroplasty for a femoral neck fracture during the study period. Forty-seven patients, who had been bed-ridden prior to injury, with a previous history of a thromboembolic event, or had expired because of causes unrelated to index surgery within one month after HFS were excluded. Seven patients in whom the occurrence of VTE could not be confirmed within one postoperative month because of follow-up loss or patients’ refusal to undergo the imaging study after discharge, were also excluded. In addition, 21 patients who had been treated with warfarin were also excluded because these patients continuously took LMWH therapy instead of warfarin preoperatively after admission. Of the remaining 539 patients, 166 took aspirin-containing compounds or other antiplatelet medication preinjury. Because their medications were discontinued on admission, these patients were not excluded. Thus, 539 patients (392 women and 147 men) were the subjects of this study (Fig 1).

The study period of 54 months included 36 months before and 18 months after the initiation of preoperative mechanical thromboprophylaxis. Patients enrolled in the current study were divided into two groups. Patients in the control group (404 patients) who underwent HFS from January 2012 to December 2014 did not receive preoperative mechanical thromboprophylaxis. Those in the study group (135 patients), who underwent HFS from January 2015 to June 2016, received preoperative mechanical thromboprophylaxis from the time of admission until surgery. Demographic data such as age, gender, body mass index (BMI), fracture site, American Society of Anesthesiologists (ASA) grade, and comorbidities were collected from the electronic patient records of our hospital. Data on comorbid medical condition were categorized based on the presence of the following conditions: cardiovascular diseases, pulmonary diseases, endocrinological diseases, neurologic diseases, psychiatric diseases, nephrological diseases, and cancer. In addition, information on the time to operation after admission, operation time, length of hospital-stay, and the method of anesthesia were collected.

All patients in both groups received combined chemical prophylaxis for 10 to 14 days and mechanical prophylaxis until discharge (for about two weeks) for VTE after HFS according to the same protocol (http://dx.doi.org/10.17504/protocols.io.jfscjne). According to our protocol, LMWH was used as a chemoprophylactic agent for VTE and the subcutaneous administration of 40 mg enoxaparin once-daily commenced 24 hours after the index surgery. All patients received medication for a total of 10 to 14 days. Mechanical thromboprophylaxis was performed using an IPCD and GCS simultaneously to enhance its efficacy both preoperatively and postoperatively in the study group and only postoperatively in the control group. This was continued until discharge. The IPCD (Kendall Express 9525 SCD: Covidien, Dublin, Ireland) used in the current study consisted of a garment fitting the calf and thigh. The garment was inflated using a pump and deflated every 35 to 45 seconds according to the venous refilling time of the patient. The inflated pressure was 45 mmHg in the ankle, 40 mmHg in the calf and 30 mmHg in the thigh. This IPCD was applied to the calf and thigh of both legs at all times. Although the actual working time of IPCD depends on the patient’s compliance, most of our patients used IPCD throughout the day and at night. Thigh-length GCS were also used at all times.

Postoperative rehabilitation that focused on early mobilization was performed according to our protocol. Tolerable weight-bearing standing and ambulation using a walker were started from two to three days after surgery. Most of the elderly patients undergoing HFS were hospitalized for approximately two weeks after surgery because the National Public Health System and private health insurance companies covered most of the cost in our country. These patients were then transferred to the Department of Rehabilitation in our hospital or affiliated rehabilitation centers or nursing facilities for continuous rehabilitation lasting approximately two to three weeks.

Postoperative routine surveillance for either DVT of lower extremities or pulmonary embolism (PE) was not performed. When patients displayed signs and symptoms corresponding to DVT or PE within one month after HFS, and when other possible causes were ruled out after evaluation, three-dimensional computed tomography (3-D CT) angiography was performed on these patients at any postoperative period in consultation with cardiovascular or pulmonary specialists. The final diagnosis of DVT and PE in these patients was made by cardiovascular and pulmonary specialists respectively, considering the correlation between clinical manifestations and findings confirmed by a radiologist based on 3-D CT angiography. After the final diagnosis was confirmed by each specialist, symptomatic DVT and PE in these patients were managed by each specialist or in consultation with them. Finally, efficacy outcomes in this study included the incidence of the following VTE events observed up to one month after surgery. Warfarin or rivaroxaban was given to all patients with confirmed VTE.

### Ethics statement

This study was approved by the local Institutional Review Board/Ethics Committee of Hallym University Sacred Heart Hospital (2016-I124), and it was conducted in compliance with the Declaration of Helsinki. As this work was a retrospective observational study, we did not obtain informed consents. Personally identifiable information of patients was encrypted and all the analyzed data were anonymized.

### Statistical analysis

The statistical package SPSS version 17 (SPSS Inc., Chicago, IL) was used for statistical analysis. Demographic and perioperative data were compared by using the Student t-test for continuous variables and the chi-square test for dichotomous variables. For comparison of the incidence of symptomatic thromboembolic events and mortality between the two groups, Fisher exact test was used owing to a small number of patients. Finally, multiple logistic regression was performed for the incidence of symptomatic VTE to determine the odds ratios (OR) and p-value adjusted for the potential confounders at p < 0.1 in univariate analyses. We performed a post hoc power analysis on our data to assess clinical relevance. Our primary outcome variable was whether there was a difference in the incidence of VTE between the two groups. Statistical significance was determined by obtaining a P < 0.05 in all the analyses.

## Results

The demographic and perioperative data of patients in the two groups are presented in Table 1. The demographic data including age, gender, fracture site, and BMI showed no difference in both groups. However, the ASA grade was significantly higher in the study group than in the control group (p = 0.016). In addition, more patients (39%) in the study group had received anticoagulant therapy because of comorbidities before injury than those (28%) in the control group (p = 0.025).

**Table 1:** Demographic and perioperative data in both two groups.

Variables		Control group (n = 404)	Study group (n = 135)	p-value
Gender				0.855
	Male	111 (28%)	36 (27%)	
	Female	293 (72%)	99 (73%)	
Fracture site				0.456
	Intertrochanter	251 (62%)	79 (59%)	
	Femur Neck	153 (38%)	56 (41%)	
Age (years)		82.2 ± 6.3	82.0 ± 5.6	0.839
Body mass index (kg/m2)		22.0 ± 3.6	21.5 ± 4.0	0.242
ASA grade				**0.016**
	II	55 (16%)	8 (6%)	
	III-IV	349 (84%)	127 (94%)	
Anticoagulant medication		114 (28%)	52 (39%)	**0.025**
	Aspirin	70	32	
	Clopidogrel	36	16	
	LMWH	2	0	
	Others	6	4	
Time to operation (days) after admission		3.2 ± 5.0	2.8 ± 3.5	0.218
Anesthesia				0.322
	General	198 (49%)	73 (54%)	
	Spinal	206 (51%)	62 (46%)	
Operation time (min)		78.9±28.6	76.7±33.8	0.762
Length of hospital stay (days)		22.7±12.4	17.4±8.5	**0.043**

Continuous variables are presented as mean±standard deviation.

ASA, American Society of Anesthesiologists; LMWH, low molecular weight heparin.

In terms of perioperative data, there were no significant differences in the time to operation, operation time, and the method of anesthesia between the two groups. However, the length of hospital-stay was significantly longer in the control group (22.7±12.4 days) than in the study group (17.4±8.5 days) (p = 0.043).

For comorbid medical conditions, the proportion of patients with comorbidities in the study group was generally greater than that of controls. In particular, more patients in the study group than in the control group had cardiovascular disease (71% vs 57%, p = 0.005) and neurologic disease (36% vs 24%, p = 0.009) (Table 2), which have been known to increase the risk of VTE. Pulmonary diseases also known as a risk factor of VTE, were present in more patients in the study group although there was no significant difference (p = 0.076).

**Table 2:** Comorbid medical diseases between the two groups.

Characteristics	Control group (n = 404)	Study group (n = 135)	p-value
Cardiovascular diseases	232 (57%)	96 (71%)	**0.005**
Pulmonary diseases	26 (6%)	15 (11%)	0.076
Endocrinologic diseases	100 (25%)	43 (32%)	0.106
Neurologic diseases	97 (24%)	48 (36%)	**0.009**
Psychotic diseases	19 (5%)	7 (5%)	0.821
Cancer	38 (9%)	16 (12%)	0.413
Nephrologic diseases	17 (4%)	9 (7%)	0.248

**Fig 2:**
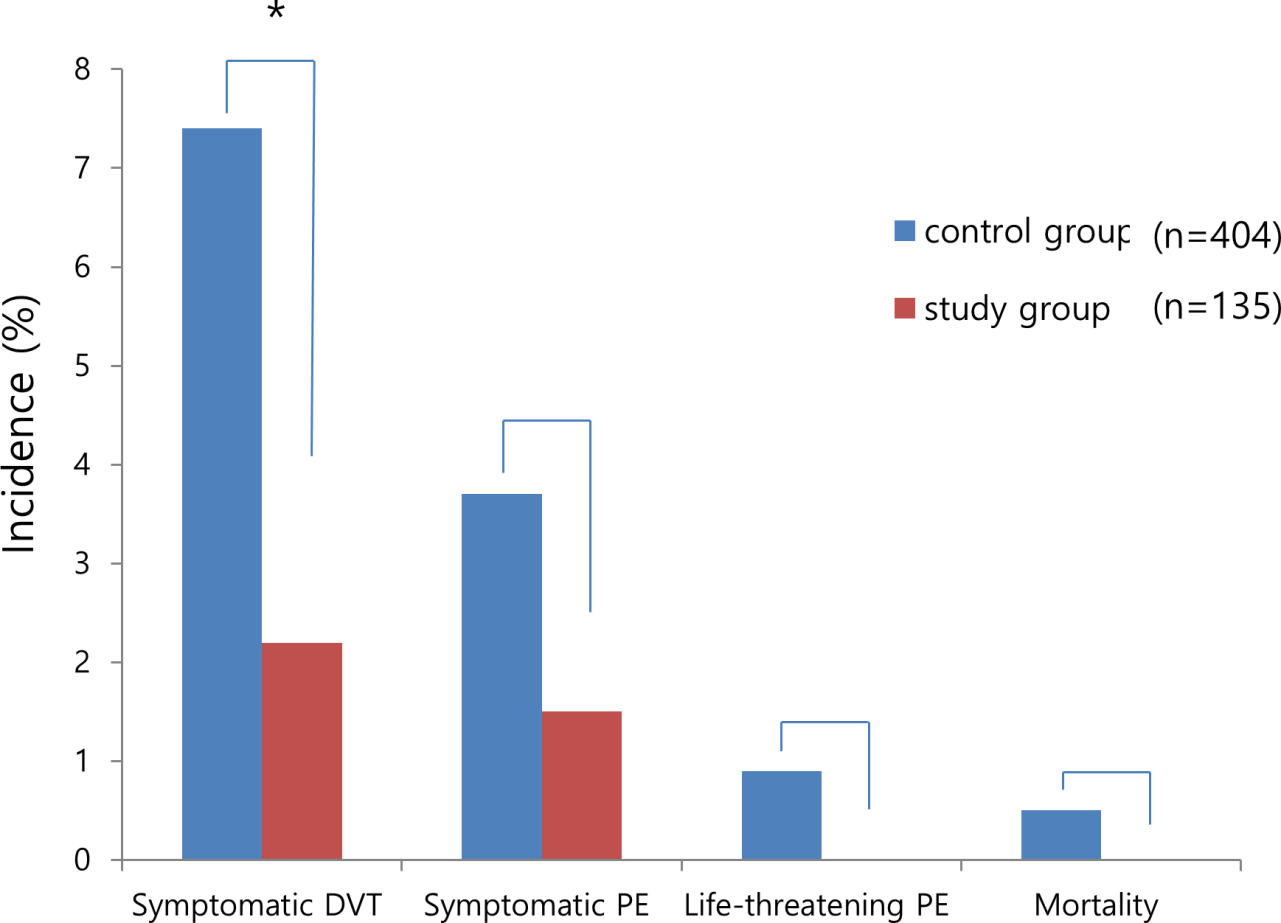
Incidence of venous thromboembolism events and mortality between the two groups (*p < 0.05).

The overall incidence of symptomatic DVT was significantly lower in the study group (2.2%) than in the control group (7.4%) (p = 0.040) although there was no significant difference in the incidence of symptomatic PE between the two groups (1.5% vs 3.7%, p = 0.215) (Fig 2). While two of three patients with symptomatic DVT in the study group showed distal DVT, one had proximal DVT (33.3%) on 3-D CT angiography. Meanwhile, 24 of 30 patients in the control group had distal DVT and the remaining six patients had proximal DVT (20.0%). There was no significant difference in the site of DVT between the two groups (p = 0.523). No life-threatening PE or mortality developed in the study group. Meanwhile, of the four patients with life-threatening PE in the control group (0.9%), two eventually expired because of serious progression (0.5%). No complications associated with IPCD and GCS used for mechanical thromboprophylaxis were observed and there were no re-operations for causes related to index surgery within one month postoperatively in this cohort.

On multiple logistic regression analysis to adjust the above outcomes to the potential confounders including ASA grade, cardiovascular disease, pulmonary disease, neurologic disease, and anticoagulant medication at p <0.1 in univariate analyses, symptomatic DVT was significantly reduced in the study group receiving preoperative mechanical prophylaxis (OR 0.28; p = 0.042), whereas there was no significant effect of preoperative mechanical prophylaxis on the prevention of symptomatic PE (Table 3).

**Table 3:** Comparison of VTE events between the two groups: Multivariate analysis*.

Events	Control group (n = 404)	Study group (n = 135)	Odds ratio (95% CI)	p-value
Symptomatic DVT	30 (7.4%)	3 (2.2%)	0.28 (0.08–0.95)	**0.042**
Symptomatic PE	15 (3.7%)	2 (1.5%)	0.39 (0.09–1.77)	0.223

*Adjusted for ASA grade, cardiovascular disease, pulmonary disease, neurologic disease, and anticoagulant

medication at p <0.1 in univariate analyses.

VTE, venous thromboembolism; CI, confidence interval; DVT, deep vein thrombosis; PE, pulmonary embolism.

## Discussion

The current findings showed that preoperative mechanical prophylaxis significantly reduced the incidence of symptomatic DVT following HFS in elderly patients over 70 years of age compared with this age group who was not receiving preoperative mechanical prophylaxis. Meanwhile, there were no significant differences in symptomatic and life-threatening PE and mortality between both groups.

The current study has several limitations. First, this is not a matched case-control study showing differences in comorbidities, preoperative implementation of anticoagulant therapy, and ASA grade between two groups. Second, we recognize the limitations of our retrospective observational study. We performed a post hoc power analysis assuming α level of 0.1 for the incidence of symptomatic VTE between the two groups, and obtained the power of 0.78 for symptomatic DVT as the primary outcome variable, and the power of 0.26 for symptomatic PE. Although our study had enough power to detect a clinically significant difference of the incidence of symptomatic DVT between the two groups, we might miss a difference related to PE between two groups because of the lower power due to the small sample size. Third, the patients may have had other comorbidities including metabolic diseases or genetic or acquired defects of the coagulation system, which might be risk factors for VTE. These comorbidities were not discussed in this study. Fourth, the present study has no data comparing the effectiveness of preoperative mechanical thromboprophylaxis with that of preoperative chemical thromboprophylaxis. Fifth, we could not completely exclude the effect of previous administered anticoagulants on postoperative VTE in patients who had received anticoagulant therapy before injury because the cessation period of this medication from the time of admission until surgery was relatively short. Therefore, we tried to overcome this limitation by performing the statistical adjustment. Lastly, the incidence of VTE following HFS was investigated within 30 days of surgery, a relatively short period postoperatively.

However, a major strength of this study is that, to our knowledge, it is the first case-control study to show the additional effectiveness of preoperative mechanical prophylaxis for preventing VTE following HFS in elderly patients. The second strength is that it focused on elderly patients aged ≥70 years with hip fracture, who were at a high risk of VTE. Moreover, elderly patients in the study group who received preoperative mechanical thromboprophylaxis were at higher risk of VTE due to having both a higher ASA grade and more comorbidities. Nevertheless, preoperative mechanical thromboprophylaxis showed significant effectiveness on the prevention of VTE in the study group. The third is that this study enrolled consecutive elderly patients aged over 70 years undergoing HFS at one hospital. The fourth is that we made relatively accurate and relevant definition and diagnosis of symptomatic VTE, confirmed by each specialist. When patients had the symptoms and signs corresponding to DVT or PE and other possible causes were ruled out after evaluation because signs and symptoms alone are not sufficiently sensitive or specific, 3D CT angiography was performed in these patients. The final diagnoses of DVT and PE were made by cardiovascular and pulmonary specialists, respectively based on clinical manifestations and radiologist’s interpretation of 3-D CT angiography. We believe that this process would lessen false positives in diagnosis of symptomatic DVT and PE and improve its accuracy. The fifth is that we could have a complete follow-up and access to all records because most patients stayed at our hospital or affiliated rehabilitation or nursing facilities for one month after HFS. Therefore, we could completely investigate complications including VTE and mortality within one month after surgery. These findings may strengthen the significance of our study.

Elderly patients undergoing HFS are at higher risk of VTE owing to associated immobility along with other risk factors [1,2,17]. The immobility due to severe hip pain during the preoperative period may cause venous stasis from the lower extremities, thus increasing the risk of VTE in these patients. Although ACCP guidelines recommend initiating LMWH preoperatively in case of delayed surgery [2], the guidelines do not provide any comparisons of effectiveness and safety between preoperative and postoperative initiation of LMWH therapy and consensus on optimal time for initiation of anticoagulation therapy is yet to be reached [18,19]. In addition, there has been a controversy regarding the effectiveness of preoperative chemical prophylaxis for preventing VTE following HFS. Thaler et al [20] reported that preoperative LMWH therapy prevented postoperative VTE efficiently. In contrast, Liu et al [5] reported that preoperative anticoagulation therapy with LMWH did not significantly reduce the risk of postoperative VTE. Perka [16] also reported no difference in the effectiveness of preoperative versus postoperative initiation of anticoagulant for thromboprophylaxis. Preoperative initiation of anticoagulation therapy can effectively reduce the risk of VTE, but may increase the risk of intraoperative and postoperative bleeding [14,15]. In addition, preoperative initiation of anticoagulant may increase the risk of intraspinal hematoma in patients placed under spinal anesthesia, [5]. Especially, in fragile elderly patients over 70 years of age undergoing HFS, bleeding risk may be as fatal as the risk of VTE [21]. Therefore, preoperative mechanical thromboprophylaxis instead of preoperative initiation of LMWH may be more beneficial and safer for further lowering the incidence of VTE without additional risk of perioperative bleeding, especially in elderly patients aged over 70 years. However, to date, there has been no report on the additional effectiveness of preoperative mechanical thromboprophylaxis on the prevention of postoperative VTE, specifically in elderly patients with only hip fracture. As shown in the current study, the additional effectiveness of preoperative mechanical prophylaxis on the prevention of VTE may be very meaningful in these elderly patients. Moreover, it is of paramount importance that there was no life-threatening PE in the study group receiving preoperative mechanical prophylaxis.

According to previous studies, age greater than 70 years, previous VTE history, family history of VTE, obesity, immobility, recent trauma or surgery, cardiovascular diseases such as hypertension and diabetes, and higher ASA grade have been known as risk factors of VTE following major orthopaedic surgery [4,22–25]. Besides, Heit et al [25] reported that neurologic disease with extremity paresis was an independent predictor of VTE recurrence owing to immobility and increased risk of hypercoagulable state. Considering the above risk factors, elderly patients with hip fracture who were enrolled in this study are at high risk of VTE because they are over 70 years of age and those in the study group receiving preoperative mechanical thromboprophylaxis were at higher risk because of higher ASA grade and more medical comorbidities. Specifically, more patients in the study group receiving preoperative mechanical thromboprophylaxis accompanied both cardiovascular diseases and neurologic diseases (p = 0.005 and p = 0.009, respectively), which are independent risk factors of VTE [22,25]. Therefore, the current finding that preoperative mechanical thromboprophylaxis significantly reduced the incidence of VTE in the study group at higher risk may provide an evidence that preoperative mechanical thromboprophylaxis provides additional effectiveness in preventing postoperative VTE in elderly patients with hip fracture.

It has been reported that the incidence of DVT after major orthopaedic surgery is low in East Asian countries including Korea, moreover the incidence of PE is much lower in those, even without any thromboprophylaxis [10,26,27]. Therefore, in our country, preoperative thromboprophylaxis had been not routinely performed in the past although postoperative thromboprophylaxis had been routinely performed in most of hospitals. Moreover, preoperative anticoagulant initiation may increase the risk of intraspinal hematoma when spinal anesthesia is performed [5], which is known to be more beneficial to elderly patients undergoing HFS in several ways. However, recently, most of orthopaedic surgeons have considered or performed routine preoperative thromboprophylaxis to further lower the incidence of VTE including fatal PE because these complications can frequently lead to considerable morbidity and mortality, especially in elderly patients with hip fracture. As part of such an effort, we have routinely performed mechanical prophylaxis using IPCD and GCS without use of anticoagulants before surgery in almost all patients. Preoperative mechanical thromboprophylaxis performed in this study has further lowered the incidences of DVT and PE. However, we believe that insignificant effect of preoperative mechanical prophylaxis on the prevention of PE is caused by very low incidence of PE and relatively small sample size considering it. Accordingly, a larger sample size will be needed in the future to verify the effect of preoperative mechanical prophylaxis on the prevention of PE.

Obviously, the current findings would contribute to the body of evidence regarding the use of preoperative mechanical thromboprophylaxis to lower the incidence of VTE further following HFS in elderly patients. However, well-controlled prospective studies or large multicenter studies are needed to draw conclusions on the efficacy of preoperative mechanical thromboprophylaxis in these patients.

In summary, preoperative mechanical thromboprophylaxis significantly reduced symptomatic DVT within one month after HFS in a high-risk group of elderly patients aged over 70 years. Therefore, preoperative mechanical prophylaxis may be of great benefit for preventing postoperative VTE without the additional risk of perioperative bleeding in elderly patients with hip fracture, who are routinely managed with combined thromboprophylaxis after HFS.
